# Survey dataset on professional's perception on site supervision and project performance

**DOI:** 10.1016/j.dib.2018.04.099

**Published:** 2018-05-04

**Authors:** David Obinna Nduka, Adegboyega Sunday Sotunbo, Idris Adedapo Ibrahim, Opeyemi Joshua, Patience E. Tunji-Olayeni, Bolatito Akinbile

**Affiliations:** a Department of Building Technology, Covenant University, Ota, Nigeria; b Department of Building, University of Lagos, Akoka, Lagos, Nigeria; c Department of Building Technology, Bells University of Technology, Ota, Nigeria

## Abstract

Effective site supervision plays an important role in construction project delivery. This situates site supervisors in taking the responsibilities of coordinating and controlling all physical aspects of the day-to-day task in construction projects. A cross-sectional design approach was adopted by administering a well-structured questionnaire to selected built environment professionals. Descriptive statistics was performed on the data obtained and are presented on figures and tables. The data was also subjected to inferential statistics using Kruskal Wallis test to analyze the perceptions of respondents on effects of site supervision of construction works on completion time and quality respectively. The significance of the analyzed data is on identifying the effects of site supervision on project completion time and quality delivery. The analyzed data will also guide project stakeholders in selecting competent personnel in executing construction projects.

**Specifications Table**

**Table:** 

Subject area	*Building Construction*
More specific subject area	*Construction Management*
Type of data	*Table, text file and figure*
How data was acquired	*Field survey*
Data format	*Raw, filtered and analyzed*
Experimental factors	*Systematic sampling of selected built environmental professionals practicing in Lagos, Nigeria*
Experimental features	*Google online form as generated and administered to selected built environment professionals in order to get their perception on the effects of supervision on construction projects.*
Data source location	*Lagos, Nigeria*
Data accessibility	*All the data are contained in this data article*

**Value of the data**

• The data reveal cogent roles of site supervisors in construction projects towards elimination of reworks, minimization of waste and ensuring conformity to standards.
• The impacts of site supervision on project accomplishment of time and quality will be a contribution provided by the data.
• The identification of the effects of site supervision will provide basics for local construction sector to equip themselves for future selection of site supervisors.
• The data will also guide academia in fulfilling their teaching requirements hence producing competent hands that can handle construction works.

## 1 Data

**Fig. 1:**
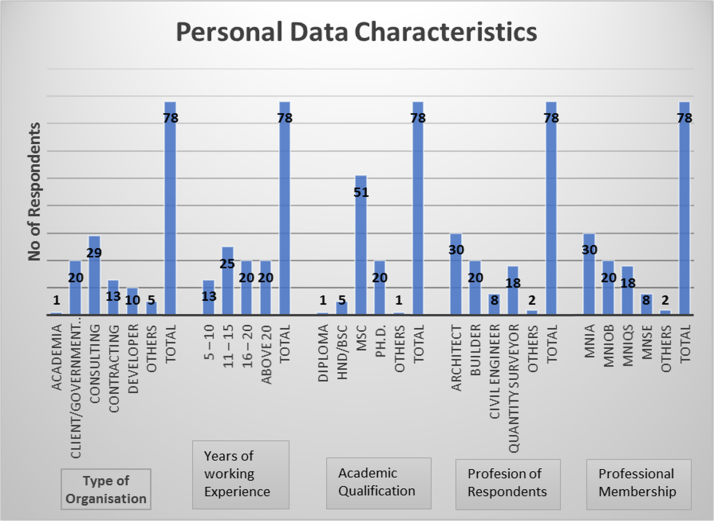
Summary of personal data of respondents.

A total of 78 online well-structured data instrument was administered to various built environment professionals comprising of Architects, Builders, Quantity Surveyors and Civil Engineers. The demographic features of the selected built environment professionals are presented in Fig. 1. The content of the designed instrument geared towards obtaining information on the perceptions of construction professionals on the effects of site supervision on construction projects. The data analysis uncovers the significant functions of site supervisors. In depth study of the data can provide insight into the roles performed by site supervisor in achieving project success of time and quality. Hypotheses were postulated which in turn lead to inferential statistics. The inferences drawn can inform the decision that inadequate site supervision can impair the timely completion and quality delivery of a construction projects. The data revealed top functions of site supervisor which include: Supervision and execution of the construction projects, Minimization of waste and elimination reworks on site, ensuring quality and standard conformity of materials and equipment, working with the purchasing procurement officer to ensure that materials are delivered in a timely manner to site to enable the contractor complete the works as scheduled and adhering to policies and procedures on site. Also possessing skills such as ability to monitor and control all assigned works, eliminating and resolving of disputes among workers and prompt reporting to the relevant project team member on the progress of and all material issues are relevant skills of site supervisor in ensuring timely completion and quality delivery of construction projects. The usefulness of this data is in its focus of project performance which has been a subject of debate among researchers and practitioners(Table 1, Table 2, Table 3, Table 4, Table 5).

**Table 1:** Roles of site supervisors in construction projects.

**S/N**	**Roles**	**Mean**	**Std. deviation**	**Rank**
1	Supervise the execution of the construction projects	2.95	.222	1
2	Minimize waste and eliminate reworks on site	2.90	.347	2
3	Ensure quality and standard conformity of materials equipment	2.90	.307	2
4	Work with the purchasing procurement officer to ensure that materials are delivered in a timely manner to site to enable the Contractor complete the Works as scheduled.	2.87	.373	4
5	Adhering to policies and procedures on site	2.87	.373	4
6	Work with the project manager during the test running and handing	2.87	.336	4
7	Undertake daily inspections of the activities on site and identify any improvements to the site safety, tidiness and performance required.	2.87	.338	4
8	Interpret working drawings regulations and codes of practice in order to direct the progress of work	2.86	.350	8
9	Prevents fines and interruptions by complying with and enforcing all relevance code	2.86	.386	8
10	Ensuring quality standards of works onsite	2.86	.350	8
11	Maintains safe secure and healthy work environment by following and enforcing standards and procedures; complying with legal regulations on safety.	2.86	.388	8
12	Ensure promptly notification of the Project Manager of any matter coming to the site supervisor's attention which could have a material adverse effect on the project performance	2.85	.397	12
13	Assign works and task to different team onsite	2.85	.363	12
14	Monitor the Contractors activities on site for compliance with the technical and quality contractual requirements.	2.85	.458	12
15	Resolve any short falls with the Contract and raise Non-Conformance reports, if required, in consultation with the project team	2.85	.397	12
16	Input incidents into the Incident Reporting System or book and be active in investigating and closing out the incident on site	2.85	.397	12
17	Provide on-site supervision of contractors and or subcontractor including the issuing of contract instructions to give direction, within delegated limits, as required.	2.83	.375	17
18	Estimate and allocate resources required for the progress of the work	2.83	.441	17
19	Identifying construction management system improvements and communicate such to the project manager	2.83	.441	17
20	Identify work practices methods and activities that can be altered or improved on site	2.83	.413	17
21	Ensure that adequate project records are maintained including, but not limited to, site diaries, contract instructions, incident reports etc.	2.82	.448	21
22	Ensure that the contractors/subcontractors receive store and use supplied/issued materials in accordance with the contractual agreement	2.81	.457	22
23	Report frequently to the relevant Project team member on the progress of the project and all material issues regarding the assigned projects.	2.81	.457	22
24	Monitor and organize assigned work on site	2.79	.543	24
25	Resolve design construction and relational problems on site	2.79	.519	24
26	Ensuring adequate communication of job expectations through planning, monitoring and appraising job contributions	2.78	.474	26
27	Evaluate communicate and implement change alteration orders	2.77	.535	27
28	Recommending compensation actions as the need arises	2.76	.539	28
29	Meet construction budget by monitoring project expenditure identifying variances; implementing corrective action and capital budget information	2.73	.574	29
30	Help to achieve project goals and objectives	1.85	.941	30

**Table 2:** Effects of adequate site supervision on project completion time.

**S/N**	**Effects**	**Mean**	**Std. deviation**	**Rank**
1	Adequate monitoring and organizing of all assigned work on site to ensure progression as planned	4.41	.673	1
2	Works are carried out to design, specifications and standards.	4.41	.813	1
3	Eliminate dispute among the workers on site	4.35	.680	3
4	Prompt materials delivery to site for the work to proceeds as planned	4.33	.816	4
5	Prompt reporting to the relevant Project team member on the progress of; and all material issues regarding the assigned projects	4.32	.712	5
6	Prompt response to complaints, queries or alterations	4.31	.827	6
7	Eliminate misunderstanding and misinterpretation of the construction documents.	4.29	.791	7
8	Avoidance of delay in any aspect of the project	4.29	.808	7
9	Ensuring that the site is safe for all workers	4.29	.841	7
10	Prompt estimating and allocating of resources required for the progress of the work	4.28	.754	10
11	Prevention of fines and interruptions from the regulatory bodies by complying with and enforcing relevance laws and codes.	4.27	.912	11
12	Effective on-site supervision of contractors and /or subcontractors including the issuing of Contract Instructions to give direction, within delegated limits, as required.	4.27	.878	11
13	Adequate monitoring and controlling of the project time performance	4.26	.801	13
14	Avoid plants and equipment total break-down that could jeopardize the progress of the works.	4.23	.755	14
15	Prompt resolution of design, construction and relational problems on site	4.19	.884	15
16	Reduce or eliminate reworks	4.16	.796	16

**Table 3:** Effects of adequate site supervision on project quality delivery.

**S/N**	**Effects**	**Mean**	**Std. deviation**	**Rank**
1	Proper management of sub-contractors by locating, evaluating, and selecting sub-contractors; monitoring and controlling performance.	4.33	.767	1
2	Usage of quality and standard materials	4.29	.913	2
3	Compliance with construction documents	4.26	.865	3
4	Provision of right tools for to carry out construction activities	4.22	.921	4
5	Effective on-site supervision of contractors and /or subcontractors including the issuing of Contract Instructions to give direction, within delegated limits, as required.	4.22	.892	4
6	Eliminate misunderstanding and misinterpretation of the construction documents.	4.22	.949	4
7	Enforcement of total quality control on site	4.21	1.00	7
8	Monitor the Contractor's activities on site and compliance with the technical and quality contractual requirements.	4.21	.888	7
9	Compliance with construction documents	4.19	.884	9
10	Prompt and proper quality testing and inspection of various construction materials	4.19	.898	9
11	Evaluating and implementing change orders	4.18	.936	11
12	Eliminate reworks	4.13	.985	12
13	Ensures quality functioning and standard conformity of materials, equipment and system	4.10	.948	13
14	Engaging experienced workers in carrying out construction activity	4.10	.831	13
15	Adhering to policies and procedures on site	4.06	.978	15

**Table 4:** Kruskal Wallis Test (Effects of site supervision on project completion time).

**S/N**	**Effects**	**Chi-Square**	**Df**	**Sig.**
1	Works are carried out to design specifications and standards	.641	2	.726
2	Reduce or eliminate reworks	5.262	2	.072
3	Eliminate misunderstanding and misinterpretation of the construction	.445	2	.801
4	Eliminate dispute among the workers on site	.155	2	.925
5	Prompt materials delivery to site for the work to proceed as planned	2.145	2	.342
6	Avoidance of delay in any aspect of the project	.522	2	.770
7	Prompt response to complaints queries or alterations	1.058	2	.589
8	Prompt resolution of design construction and relational problem	2.162	2	.339
9	Prompt reporting to the relevant Project team member on the prog	.713	2	.700
10	Effective on-site supervision of contractors and or subcontract	.355	2	.837
11	Adequate monitoring and organizing of all assigned work onsite	.295	2	.863
12	Adequate monitoring and controlling of the project time performance	4.811	2	.090
13	Prompt estimating and allocating of resources required for the p	.665	2	.717
14	Prevention of fines and interruptions from the regulatory bodies	1.414	2	.493
15	Avoid plants and equipment total breakdown that could jeopardize	1.193	2	.551
16	Ensuring that the site is safe for all workers	.295	2	.863

a. Kruskal Wallis Test.

b. Grouping Variable: Years of working experience in the construction industry.

**Table 5:** Kruskal Wallis Test (Effects of site supervision on project quality delivery).

**Effects**	**Chi-Square**	**Df**	**Sig.**
Engaging experienced workers in carrying out construction activities	4.521	2	.104
Eliminate reworks	7.413	2	.025
Eliminate misunderstanding and misinterpretation of the construction	6.624	2	.036
Usage of quality and standard materials	3.425	2	.180
Proper management of subcontractors by locating, evaluating and	3.325	2	.190
Compliance with construction documents	7.266	2	.026
Compliance with construction documents	3.951	2	.139
Provision of right tools for to carry out construction activities	2.051	2	.359
Effective on-site supervision of contractor sand or subcontract	2.236	2	.327
Ensures quality functioning and standard conformity of material	1.032	2	.597
Monitor the Contractors activities on site and compliance with	5.666	2	.059
Adhering to policies and procedures onsite	.649	2	.723
Evaluating and implementing change orders	2.218	2	.330
Prompt and proper quality testing and inspection of various construction works	2.003	2	.367
Enforcement of total quality control on site	.177	2	.915

a. Kruskal Wallis Test

b. Grouping Variable: Years of working experience in the construction industry.

## 2 Experimental design, materials and methods

The collected data was based on the survey of selected construction practitioner's perception on the effects of site supervision on project completion time and quality delivery. Several literatures have reported similar accounts of the subject matter and can be found in [1], [2], [3], [4], [5], [6], [7], [8], [9], [10]. The population of the data are built environment professions practicing in Lagos State, Nigeria as obtained from their respective membership directory. Lagos State was selected because of her unprecedented commercial, economic and huge construction activities in Nigeria. The population are members of professional bodies of the state chapters of Nigerian Institute of Building (NIOB), Nigeria Institute of Architects (NIA), Nigerian Institute of Quantity Surveying (NIQS) and Nigerian Society of Engineers (NSE) (Civil Engineers). Systematic sampling technique was used in selecting 300 respondents from the population wherein the instrument was administered and returned via online google form. A total of 86 (28.7%) online questionnaires were returned out of which 8 were invalid. Evidence from literatures show that studies [11], [12], [13], [14] have utilized survey design in assessing the impact of site supervision on construction projects. The responses were rated on a five-point Likert scale (1 = strongly disagreed, 2 = disagreed, 3 = uncertain, 4 = agreed and 5 = strongly agreed) for effects of site supervision of construction works on completion time while a three-point Likert scale (1 = no, 2 = rarely, 3 = yes) was used for roles of site supervisors. The data collected were coded and entered into the Statistical Package for Social Sciences (SPSS) IBM 21 for analysis. Descriptive statistical tools such as frequency, percentage, mean and ranking and Kruskal-Wallis test tool (inferential tool) were used to present the data. These data are elemental part of the factors leading to project success or failure. Further studies could be conducted on comparing the effects of site supervision of construction projects delivery: a case of indigenous and foreign (multi-national) contractors.
